# The comparison of the anatomic stage and pathological prognostic stage according to the AJCC 8th edition for the prognosis in Japanese breast cancer patients: data from a single institution

**DOI:** 10.1007/s12282-020-01116-w

**Published:** 2020-05-29

**Authors:** Eriko Tokunaga, Hideki Ijichi, Wakako Tajiri, Takanobu Masuda, Katsumi Takizawa, Hiroki Ueo, Chinami Koga, Junko Tanaka, Yoshiaki Nakamura, Shinji Ohno, Kenichi Taguchi, Masahiro Okamoto

**Affiliations:** 1 Department of Breast Oncology, National Hospital Organization Kyushu Cancer Center, 3-1-1 Notame, Minami-ku, Fukuoka, 811-1395 Japan; 2 Departments of Pathology, National Hospital Organization Kyushu Cancer Center, 3-1-1 Notame, Minami-ku, Fukuoka, 811-1395 Japan; 3 Breast Cancer Center, Cancer Institute Hospital, 3-8-31 Ariake, Koutou-ku, Tokyo, 135-8550 Japan

**Keywords:** Breast cancer, TNM, AJCC, Pathological prognostic stage

## Abstract

**Background:**

The TNM system, which reflects the anatomical extent of disease, was used for stage definition. In the recently published AJCC 8th edition, the new staging system of the clinical and pathological prognostic stage, which incorporates biological factors, is introduced.

**Patients and methods:**

A total of 2622 patients with primary breast cancer at stage I–III were included in this study. The anatomic stage (aStage) and the pathological prognostic stage (ppStage) for each case were determined according to the definition of the AJCC 8th edition, and the influence of these stages on the prognosis was compared.

**Results:**

The stage distributions of aStage and ppStage were as follows: aStage, stage IA (54.8%), IB (1.1%), IIA (26.1%), IIB (9.2%), IIIA (5.6%), IIIB (0.1%), and IIIC (3.1%); and ppStage, stage IA (66.6%), IB (13.1%), IIA (11.1%), IIB (3.2%), IIIA (3.3%), IIIB (1.4%), and IIIC (1.2%). Compared with the aStage, the ppStage stayed the same in 1710 patients (65.2%), was downstaged in 778 patients (29.7%), and was upstaged in 134 patients. The pathological tumor size (pT2) and lymph node metastasis (pN1) were associated with downstaging, and histological grade 3 was associated with upstaging. ER positivity, PgR positivity, and HER2-positivity were significantly associated with downstaging, and the TN subtype was associated with upstaging. Both the aStage and ppStage were significantly associated with the prognosis; however, the Kaplan–Meier curves for the relapse-free survival (RFS), distant recurrence-free survival (DRFS), and overall survival were better stratified by the ppStage.

**Conclusion:**

The ppStage reflects the prognosis of patients with early breast cancer more accurately than the aStage.

## Introduction

An improved understanding of the breast cancer biology has greatly changed the therapeutic strategies for both early and advanced breast cancer. The evaluations of the expression of estrogen receptor (ER) and progesterone receptor (PgR) and human epidermal growth factor 2 (HER2) status are necessary to choose the most appropriate treatment. In addition, a number of factors, including the tumor grade, Ki67 labeling index, and results of multigene assays, such as Oncotype Dx, are considered when determining the adjuvant therapy for early breast cancer [1, 2].

The TNM (primary tumor [T], regional lymph node [N], and distant metastases [M]) staging system by American Joint Committee on Cancer (AJCC) began in 1959 [3]. Since then, it has been employed worldwide, including in Japan, to manage breast cancer. Recently, the 8th edition of AJCC staging system was published [4], showing substantial changes from the 7th edition. The most critical change is the introduction of the prognostic stage (clinical prognostic stage and pathological prognostic stage).

The prognostic stage incorporates the biological factors, such as the tumor grade, ER and PgR expression and HER2 overexpression and/or amplification. If the OncotypeDx® test is performed in cases with a T1N0M0 or T2N0M0 cancer that is ER + /HER2-, the recurrence score (RS) is used to determine the pathological prognostic stage (ppStage) [4]. The ppStage is based on all clinical information, biomarker data, and findings from surgery and resected tissue, and it is expected to reflect the outcomes of patients with breast cancer more accurately than the anatomic stage (aStage).

Since the publication of the AJCC 8th edition, some institutions have reported the validation of the new AJCC 8th edition of the staging system [5–9]. However, the reported differences in the stage between the aStage and ppStage, as well as the prognosis with each stage, have varied among reports.

In the present study, we retrospectively compared the two clinical staging systems of the aStage and ppStage established by the 8th edition of the AJCC staging system and analyzed the clinical significance of the prognostic staging system proposed in the treatment of early breast cancer.

## Patients and methods

### Patient population

A total of 2622 patients with primary breast cancer at stage I–III, who underwent surgery without neoadjuvant systemic therapy in the Department of Breast Oncology, National Hospital Organization Kyushu Cancer Center between 2003 and 2016 were included in this study. The clinical data were obtained from the patients’ medical records. Adjuvant treatment had been determined considering various factors, according to the clinical guidelines and the recommendations of the expert panel at that time as well as based on the clinical condition and preferences of the patients. Written informed consent was obtained from all of the patients before collecting the tissue samples. This study was approved by the institutional review board in our hospital.

### Pathological examinations

All pathological examinations were performed by the experienced pathologists in our hospital. The expression of ER and PgR was regarded as positive if their nuclear expression was ≥ 1%. The HER2 status was evaluated according to the recommendation of ASCO/CAP; HER2 score of 0 and 1 was defined as negative, while score of 3 was defined as positive. For HER2 score 2, amplification of HER2 was confirmed by in situ hybridization methods (FISH or DISH) [10].

### Stage classification

The aStage and ppStage for each case were determined according to the definition of the 8th AJCC TNM stage group tables [11]. For the definition of primary tumor (T) and regional lymph nodes (N), pathological T (pT) and pathological N (pN) were used in this study, respectively.

### Statistical analyses

The statistical analyses were performed using the JMP software package, version 14.0 (SAS Institute Inc., Cary, NC, USA). The associations between the clinicopathological characteristics were assessed using *χ*^2^ tests. The relapse-free survival (RFS) was defined as the time from surgery to the first breast cancer event, including loco-regional recurrence, distant metastasis or a new cancer in the contralateral breast. The distant recurrence-free survival (DRFS) was defined as the time from the date of curative surgery to the detection of distant recurrence. The overall survival (OS) was defined as the time from the date of curative surgery to death. Survival curves were plotted using the Kaplan–Meier method, and the association between the survival and each variable was determined by the log-rank test. Differences were considered to be significant at *p* < 0.05.

## Results

### Patients’ characteristics

In total, 2622 patients with primary breast cancer who underwent curative surgery were included in this study. The clinicopathological characteristics of these patients are described in Table 1. The median age of the patients was 58 years (range 19–91). ER, PgR, and HER2 were positive in 2136 (81.5%), 1790 (68.3%) and 420 patients (16.0%), respectively. Positivity of hormone receptor (HR+) was defined as ER+ and/or PR+. In terms of tumor subtypes determined by HR and HER2, HR+ /HER2− subtypes were observed in 2039 patients (77.8%), HR+ /HER2+ in 154 (5.9%), HR− /HER2+ 176 (6.7%), and HR−/HER2− (triple negative; TN) in 253 (9.6%). Adjuvant endocrine therapy was administrated to 92.6% (2030/2193) of the patients with HR+ tumors (2030/2193). Adjuvant chemotherapy was performed for 1032 (39.4%) of the patients. Trastuzumab was used for only 150 of the 450 patients with HER2+ tumors. In Japan, adjuvant trastuzumab was approved in 2008, so few patients with HER2+ tumors received adjuvant trastuzumab before 2008. Among the 272 patients who underwent surgery between 2008 and 2016, 146 (53.6%) were treated with adjuvant trastuzumab.

**Table 1:** Clinicopathological characteristics of the patients

Factors	*n* = 2622
Age, median, range	58 (19–91)
Age groups (years)	
≤ 39	180 (6.9)
40–49	584 (22.3)
50–59	698 (26.7)
60–69	711 (27.1)
≥ 70	444 (17.0)
Histology	
IDC	2405 (91.7)
ILC	101 (2.9)
Mucinous	83 (3.2)
Others	33 (1.2)
Histological grade	
1	595 (22.7)
2	1331 (50.8)
3	696 (26.5)
ER	
Negative	486 (18.5)
Positive	2136 (81.5)
PgR	
Negative	832 (31.7)
Positive	1790 (68.3)
HER2	
Negative	2202 (84.0)
Positive	420 (16.0)
Subtype	
HR+/HER2−	2039 (77.8)
HR+/HER2+	154 (5.9)
HR−/HER2+	176 (6.7)
HR−/HER2−	253 (9.6)
Adjuvant therapy	
Endocrine therapy	2030 (77.4)
Chemotherapy	1032 (39.4)
Trastuzumab	150 (5.7)
Anatomic stage (aStage)	
IA	1437 (54.8)
IB	29 (1.1)
IIA	684 (26.1)
IIB	241 (9.2)
IIIA	147 (5.6)
IIIB	3 (0.1)
IIIC	81 (3.1)
Pathological prognostic stage (ppStage)	
IA	1747 (66.6)
IB	346 (13.1)
IIA	292 (11.1)
IIB	83 (3.2)
IIIA	88 (3.3)
IIIB	37 (1.4)
IIIC	31 (1.2)
Stage change between aStage and ppStage	
Same	1710 (65.2)
Down-stage	778 (29.7)
Up-stage	134 (5.1)

*aStage* anatomic stage, *ppStage* pathological prognostic stage, *IDC* invasive ductal carcinoma, *ILC* invasive lobular carcinoma, *ER* estrogen receptor, *PgR* progesteronr receptor, *HER2* human epidermal growth factor receptor 2, *HR* hormone receptor

The stage distribution of aStage was as follows: stage IA (54.8%), IB (1.1%), IIA (26.1%), IIB (9.2%), IIIA (5.6%), IIIB (0.1%), and IIIC (3.1%). The stage distribution of ppStage was as follows: stage IA (66.6%), IB (13.1%), IIA (11.1%), IIB (3.2%), IIIA (3.3%), IIIB (1.4%), and IIIC (1.2%). Thus, the proportion of patients in stage IA and IB was higher for the ppStage than for the aStage.

### Changes in the stage between the aStage and the ppStage

The relationships between the aStage and ppStage are shown in Table 2. BCs with an aStage of stage IA (*n* = 1437) were redistributed to a ppStage of stage IA (*n* = 1336, 93.0%), stage IB (*n* = 99, 6.9%), and stage IIIA (*n* = 2, 0.1%). More than half of the BCs with stage IIA of aStage (*n* = 684) were redistributed to a ppStage of stage IA (*n* = 350, 51.2%), and many of the BC with an aStage of IIB (*n* = 241) was downstaged to stage IA (*n* = 27, 11.2%), stage IB (*n* = 102, 42.3%), and stage IIA (*n* = 36, 14.9%, Table 2) for the ppStage. In total, compared with aStage, the ppStage was the same in 1710 patients (65.2%), and downstaged in 778 patients (29.7%) and upstaged in 134 patients (5.1%, Tables 1 and 2). The relationships between the clinicopathological characteristics and the changes in the stage from aStage to ppStage are shown in Table 3. The pathological tumor size (pT2) and lymph node metastasis (pN1) were associated with downstaging, and histological grade 3 was associated with upstaging. ER positivity, PgR positivity, and HER2-positivity were significantly associated with downstaging, and the TN subtype was associated with upstaging. These associations were expected to some degree based on the definition of ppStage.

**Table 2:** Relationships between the aStage and the ppStage by AJCC 8th edition

			aStage														Total
			IA		IB		IIA		IIB		IIIA		IIIB		IIIC		
ppSatge																	
IA	*n*	1336		28		350		27		2		1		3		1747	
	%	93.0%		96.6%		51.2%		11.2%		1.4%		33.3%		3.7%		66.6%	
IB	*n*	99		1		81		102		63		0		0		346	
	%	6.9%		3.4%		11.8%		42.3%		42.9%		0%		0%		13.2%	
IIA	*n*	0		0		252		36		4		0		0		292	
	%	0%		0%		36.8%		14.9%		2.7%		0%		0%		11.1%	
IIB	*n*	0		0		1		60		22		0		0		83	
	%	0%		0%		0.1%		24.9%		15.0%		0%		0%		3.2%	
IIIA	*n*	2		0		0		16		40		1		27		86	
	%	0.1%		0%		0%		6.6%		27.2%		33.3%		33.3%		3.3%	
IIIB	*n*	0		0		0		0		5		1		31		37	
	%	0%		0%		0%		0%		3.4%		33.3%		38.3%		1.4%	
IIIC	*n*	0		0		0		0		11		0		20		31	
	%	0%		0%		0%		0%		7.5%		0%		24.7%		1.2%	
Total	*n*	1437		29		684		241		147		3		81		2622	
	%	54.8%		1.1%		26.1%		9.2%		5.6%		0.1%		3.1%		100.0%	

*aStage* anatomic stage, *ppStage* pathological prognostic stage

**Table 3:** Relationships between the clinicopathological characteristics and the stage changes

Factors	Down-stage (*n* = 778)	Same stage (*n* = 1710)	Up-stage (*n* = 134)	*p* value
pT				
1	232 (29.8)	1413 (82.6)	105 (78.4)	< 0.0001
2	489 (62.9)	273 (16.0)	17 (12.7)	
3-	57 (7.3)	24 (1.4)	12 (9.0)	
pN				
0	283 (26.4)	1536 (89.8)	104 (77.6)	< 0.0001
1 mic	43 (5.5)	2 (0.1)	1 (0.8)	
1	314 (40.4)	115 (6.7)	22 (16.4)	
2	77 (9.9)	37 (2.2)	7 (5.2)	
3	61 (7.8)	20 (1.2)	0 (0)	
Histological grade				
1	150 (19.3)	445 (26.0)	0 (0)	< 0.0001
2	440 (56.6)	842 (49.2)	49 (36.6)	
3	188 (24.2)	423 (24.7)	85 (63.3)	
ER				
Negative	27 (3.5)	332 (19.4)	127 (94.8)	< 0.0001
Positive	751 (96.5)	1378 (80.6)	7 (5.2)	
PgR				
Negative	47 (6.0)	656 (38.4)	129 (96.3)	< 0.0001
Positive	731 (94.0)	1052 (61.6)	5 (3.7)	
HER2				
Negative	669 (86.0)	1400 (81.9)	133 (99.3)	< 0.0001
Positive	109 (14.0)	310 (18.1)	1 (0.7)	
Subtype				
HR+/HER2−	678 (87.2)	1354 (79.2)	7 (5.2)	< 0.0001
HER2+	100 (12.9)	230 (13.5)	0 (0)	
TN	0 (0)	126 (7.4)	127 (94.8)	

*HR* hormone receptor

### The prognosis according to the aStage and ppStage

**Fig. 1:**
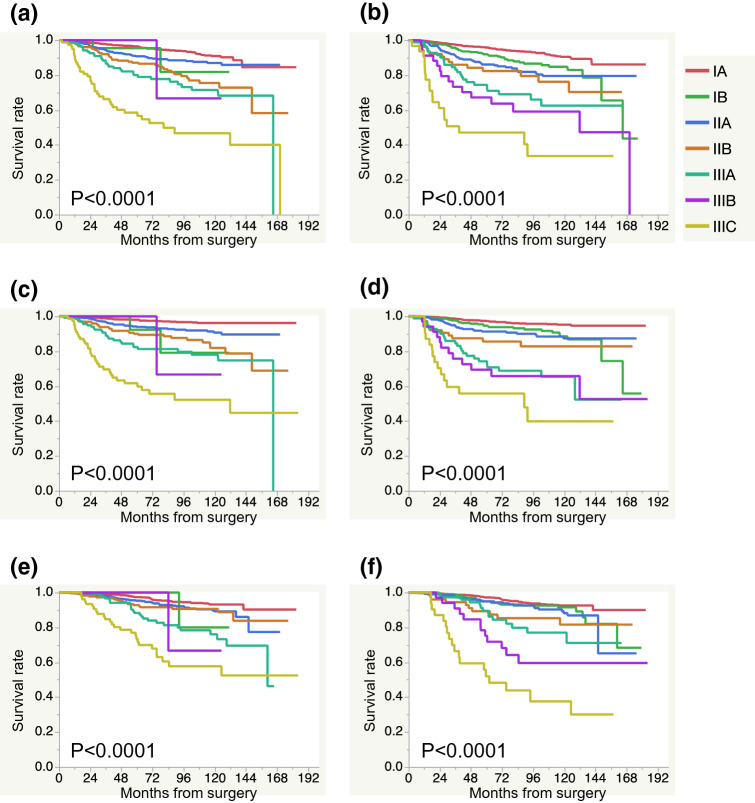
The relapse-free survival (RFS), distant recurrence-free survival (DRFS) and overall survival (OS) according to the stage determined by anatomical stage (aStage) and pathological prognostic stage (ppStage). **a** RFS by aStage, **b** RFS by ppStage, **c** DRFS by aStage, **d** DRFS by ppStage, **e** OS by aStage, **f** OS by ppStage. The number in each stage was as follows: aStage IA *n* = 1437, IB *n* = 29, IIA *n* = 684, IIB *n* = 241, IIIA *n* = 147, IIIB *n* = 3, IIIC *n* = 81. ppStage IA *n* = 1747, IB *n* = 346, IIA *n* = 292, IIB *n* = 83, IIIA *n* = 86, IIIB *n* = 37, IIIC *n* = 31

The prognosis according to the aStage and ppStage is shown in Fig. 1. Both the aStage and ppStage were significantly associated with the prognosis, in terms of the RFS (Fig. 1a, b), DRFS (Fig. 1c, d), and OS (Fig. 1e, f). However, all Kaplan–Meier curves were better stratified by the ppStage (Fig. 1b, d, f) than by the aStage. The RFS, DRFS, and OS at 5 and 10 year after surgery for each stage by the aStage and ppStage groups are shown in Table 4. All of the RFS, DRFS, and OS values were divided more clearly by the ppStage than by the aStage.

**Table 4:** The RFS, DRFS, and OS at 5 and 10 years stratified by aStage and ppStage

Stage	Number	RFS (%)				DRFS (%)				OS (%)			
		5 years	95% CI	10 years	95% CI	5 years	95% CI	10 years	95% CI	5 years	95% CI	10 years	95% CI
aStage													
IA	1437	96.5	95.2–97.4	91	88.4–93.0	98.1	97.1–98.8	96.2	94.6–97.4	97.4	96.2–98.2	93.2	90.9–94.9
IB	29	95.5	73.9–99.4	81.8	44.2–96.2	92.3	60.9–98.9	79.1	42.6–95.1	100	–	80	30.9–97-3
IIA	684	90.8	88.1–93.0	87	83.3–90.0	94	91.6–95.7	90.7	89.1–93.3	95.3	93.2–96.8	89.2	85.3–92.2
IIB	241	87.1	81.7–91.1	75.5	67.3–82.3	90	85.0–93.5	81.9	73.6–88.0	92.8	88.1–95.7	90.6	85.3–94.2
IIIA	147	80	71.8–86.3	71.5	61.5–79.8	82.4	74.4–88.3	78	68.7–85.1	88.3	80.7–93.1	76.1	65.5–84.1
IIIB	3	100	–	66.7	15.4–95.7	100	–	66.7	15.4–95.7	100	–	66.7	15.4–95.7
IIIC	81	58.5	46.7–69.4	46.7	33.9–59.9	59.8	49.7–72.4	52.5	39.1–65.0	73.5	61.6–82.7	57.7	44.0–70.3
ppStage													
IA	1747	95.8	94.6–96.8	90.5	88.1–92.4	97.3	96.3–98.1	95.3	93.6–96.5	97.2	96.1–98.0	92.6	90.5–94.2
IB	346	92	88.2–94.6	84.8	79.1–89.1	94.7	91.3–96.8	88.6	82.6–92.7	95	91.6–97.0	91.5	86.8–94.6
IIA	292	86.1	81.1–89.9	79.5	73.3–84.6	91.3	87.0–94.3	87.4	81.7–91.5	94.9	91.2–97.1	88.2	82.0–92.4
IIB	83	82.3	71.2–89.8	76.2	62.5–86.0	85.6	75.1–92.1	82.8	70.9–90.5	89.3	79.1–94.9	81.6	67.8–90.3
IIIA	86	72.7	61.3–81.8	62.5	48.8–74.5	74.3	61.0–81.7	65.4	52.2–76.6	89.5	79.6–94.5	77	63.7–86.5
IIIB	37	67.2	49.9–80.8	59.1	41.1–75.0	69.4	51.9–82.7	65.8	48.0–80.0	75.2	57.7–87.1	59.6	41.0–75.8
IIIC	31	47.1	30.2–64.7	33.6	16.9–55.7	55.8	37.7–72.5	39.9	20.4–63.1	52	34.2–69.3	37.6	20.7–58.2

*aStage* anatomic stage, *ppStage* pathological prognostic stage, *CI* confidence interval, *RFS* recurrence-free survival, *DRFS* distant recurrence-free survival, *OS* overall survival

### Relationships between the tumor subtypes and the prognosis according to the stage determined by aStage and ppStage

**Fig. 2:**
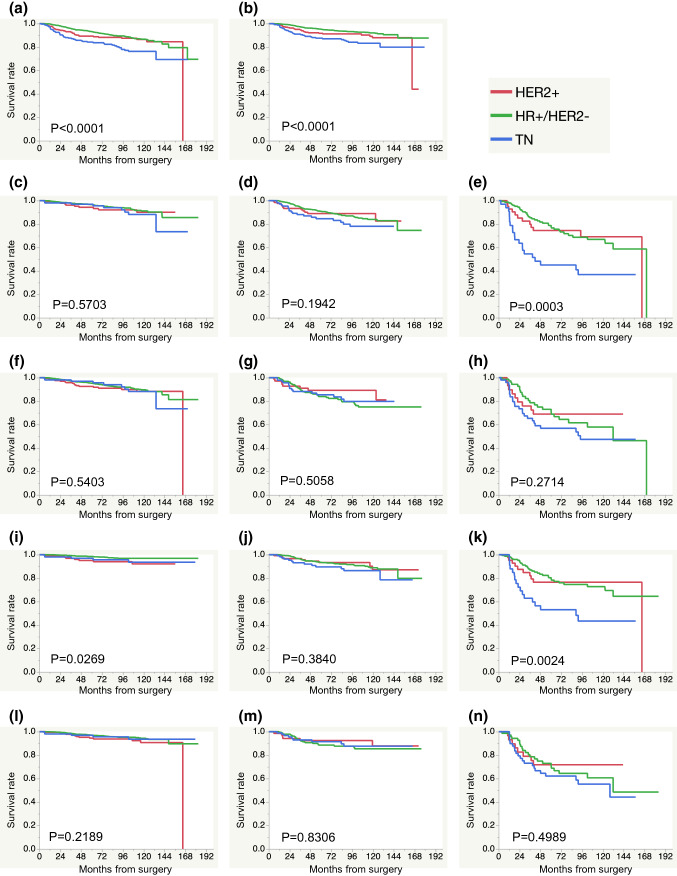
Relationships between the tumor subtypes and relapse-free survival (RFS) and distant recurrence-free survival (DRFS) according to the anatomical stage (aStage) and pathological prognostic stage (ppStage). **a** RFS of the whole cohort, **b** DRFS of the whole cohort, **c** RFS in the aStage I, **d** RFS in the aStage II, **e** RFS in the aStage III, **f** RFS in the ppStage I, **g** RFS in the ppStage II, **h** RFS in the ppStage III, **i** DRFS in the aStage I, **j** DRFS in the aStage II, **k** DRFS in the aStage III, **l** DRFS in the ppStage I, **m** DRFS in the ppStage II, **n** DRFS in the ppStage III. The number of each subtype in each stage was as follows: HR+/HER2− in aStage I *n* = 1207, in aStage II *n* = 678, in aStage III *n* = 154, HER2+ in aStage I *n* = 153, in aStage II *n* = 133, in aStage III *n* = 44, TN in aStage I *n* = 106, in aStage II *n* = 114, in aStage III *n* = 33, HR+/HER2− in ppStage I *n* = 1762, in ppStage II *n* = 203, in ppStage III *n* = 74, HER2+ in ppStage I *n* = 225, in ppStage II *n* = 74, in ppStage III *n* = 31, TN in ppStage I *n* = 106, in ppStage II *n* = 98, in ppStage III *n* = 49

The RFS and DRFS of each tumor subtype were analyzed. In the total cohort, the prognosis of the TN subtype was significantly worse than that of the other subtypes in terms of both the RFS and DRFS (Fig. 2a, b). The RFS and DRFS were then analyzed for each stage (stage I, II, and III) according to the aStage or ppStage. The RFS of the TN subtype was significantly poorer for aStage III than for the other subtypes, although there were no significant differences in the RFS among the subtypes in aStage I and II (Fig. 2c–e). In contrast, there were no significant differences in the RFS among subtypes in ppStage I–III (Fig. 2f–h). In terms of the DRFS, the value in the HR+/HER2− subtype was significantly better for aStage I than for the other subtypes (Fig. 2i), and that of the TN subtype was significantly poorer in for aStage III than for the HR+/HER2− and HER2+ subtypes (Fig. 2k). However, there were no significant differences in the DRFS among subtypes in ppStage I–III (Fig. 2l–n). Thus, according to the aStage, the prognosis differs among subtypes even in for those with the same stage. However, according to the ppStage, the difference in the prognosis for those in the same stage was not significant among the subtypes.

## Discussion

Recent advances in research concerning the subtypes and biological characteristics of breast cancer have greatly changed the treatment strategies for breast cancer. In addition to the tumor size and the lymph node metastasis, many biological factors, including the tumor grade, growth activity (Ki67 index), expression of HR and HER2 status and multigene assays, are taken into consideration when deciding on adjuvant treatment strategies.

While the anatomic TNM classifications (aStage) remain the basis of stage classification in the AJCC 8th edition, a new staging system, known as the clinical and pathologic prognostic stage (ppStage) was adopted [4]. In this new staging system, the ER, PgR, HER2, and tumor grade were incorporated into the prognostic stage definition. Since the publication of the AJCC 8th edition, several studies have compared the 7th and 8th edition of the AJCC staging system [5–8]. Many noted that the new prognostic stage provides a more powerful tool for predicting breast cancer outcomes than the aStage. For example, Lee et al. reported that, according to the aStage, the 5-year OS of patients with stage III HR+/HER2− subtype was superior to that of those with a stage II TN subtype. However, according to the clinical prognostic stage, both the 5-year disease-free survival (DFS) and OS rates of patients with stage II TN subtype were higher than those of patients with stage III HR+/HER2− subtype [8]. Both the frequencies and the impact on the prognosis on the changes from the anatomic stage to either the clinical or pathological prognostic stage were found to differ markedly among studies.

In the present study, we used the ppStage instead of the clinical prognostic stage because we believe that the ppStage reflects the prognosis more accurately than the clinical prognostic stage. Compared to the aStage, 778 patients (29.7%) were downstaged and 134 patients (5.1%) were upstaged using the ppStage. Especially, with the ppStage, the number of patients with stage IA and IB disease were markedly greater than those with the aStage. ER-, PgR- and HER2-positivity, the tumor size of pT2, and the nodal status of pN1 were significantly associated with the downstaging, while histological grade 3 and TN subtype were associated with the upstaging. These results were similar to those from a recent study conducted in Korea [12].

In the current study, we confirmed the impact of the tumor subtypes on the prognosis in the same stage by aStage and ppStage. As expected, the RFS and DRFS were significantly poorer for the TN subtypes by aStage than for other subtypes. In contrast, according to the ppStage, there were no differences in the RFS or DRFS among subtypes for stage I, II or III. The impact of the prognostic stage on the prognosis seems to differ among subtypes and studies. Prognostic staging provides no better discriminatory ability concerning the prognosis than anatomical staging in the TN subtype [13]. In contrast, however, in HER2-positive breast cancer in the ShortHER trial, the utility of the prognostic stage was validated [14].

The merit of this study is that these data are form a single institution with high-quality follow-up and updated clinical data. However, these are several limitations associated with our study as well. All data are retrospective, and the duration of the follow-up was not sufficient. Furthermore, the details of adjuvant treatments were not included in our study. In our study, we aimed to evaluate the ppStage, because the ppStage reflects the prognosis more precisely than the clinical prognostic stage. Therefore, the patients who had received neoadjuvant systemic therapy were excluded. The rate of trastuzumab usage for the patients with HER2+ tumors was less than the current standard rate, because many patients in the present study were treated in the era before the approval of adjuvant trastuzumab. These potential biases might have affected the results of the present study. The new staging system ppStage places greater emphasis on the tumor grade and tumor subtypes than the aStage. However, the tumor burden, such as the tumor size and nodal involvement, is also important to consider when treating breast cancer patients. The proportion of patients diagnosed at a very early stage was much higher in this study than in previous studies. The medical systems, screening systems, and medical insurance systems differ markedly among countries and regions. Therefore, it is important to evaluate the clinical significance of the ppStage established in the 8th edition of the AJCC staging system in other countries and regions as well. A long follow-up will also be important for evaluating the clinical significance of the new staging system.

## References

[1] Burstein HJ, Curigliano G, Loibl S, Dubsky P, Gnant M, Poortmans P (2019). Estimating the benefits of therapy for early stage breast cancer the St Gallen international consensus guidelines for the primary therapy of early breast cancer 2019. Ann Oncol..

[2] Curigliano G, Burstein HJ, Winer EP, Gnant M, Dubsky P, Loibl S (2017). De-escalating and escalating treatments for early-stage breast cancer: the St. Gallen International Expert Consensus Conference on the Primary Therapy of Early Breast Cancer 2017. Ann Oncol..

[3] Amin MB, Greene FL, Edge SB, Compton CC, Gershenwald JE, Brookland RK (2017). The Eighth Edition AJCC Cancer Staging Manual: Continuing to build a bridge from a population-based to a more "personalized" approach to cancer staging. CA Cancer J Clin..

[4] Hortobagyi GN, Connolly JL, D’Orsi CJ, Edge SB, Mittendorf EA, Rugo HS, Amin MB, Edge SB, Greene FL (2017). Breast. AJCC cancer Staging Manual.

[5] Kim I, Choi HJ, Ryu JM, Lee SK, Yu JH, Kim SW (2018). Prognostic validation of the American Joint Committee on Cancer 8th staging system in 24,014 Korean patients with breast cancer. J Breast Cancer.

[6] Kim JY, Lim JE, Jung HH, Cho SY, Cho EY, Lee SK (2018). Validation of the new AJCC eighth edition of the TNM classification for breast cancer with a single-center breast cancer cohort. Breast Cancer Res Treat..

[7] Kurundkar A, Gao X, Zhang K, Britt JP, Siegal GP, Wei S (2018). Comparison of AJCC anatomic and clinical prognostic stage groups in breast cancer: analysis of 3322 cases from a single institution. Clin Breast Cancer..

[8] Lee SB, Sohn G, Kim J, Chung IY, Lee JW, Kim HJ (2018). A retrospective prognostic evaluation analysis using the 8th edition of the American Joint Committee on Cancer staging system for breast cancer. Breast Cancer Res Treat..

[9] Shao N, Xie C, Shi Y, Ye R, Long J, Shi H (2019). Comparison of the 7th and 8th edition of American Joint Committee on Cancer (AJCC) staging systems for breast cancer patients: a surveillance, epidemiology and end results (SEER) analysis. Cancer Manag Res..

[10] Wolff AC, Hammond ME, Hicks DG, Dowsett M, McShane LM, Allison KH (2013). Recommendations for human epidermal growth factor receptor 2 testing in breast cancer: American Society of Clinical Oncology/College of American Pathologists clinical practice guideline update. J Clin Oncol.

[11] Giuliano AE, Connolly JL, Edge SB, Mittendorf EA, Rugo HS, Solin LJ (2017). Breast cancer-major changes in the American Joint Committee on Cancer eighth edition cancer staging manual. CA Cancer J Clin..

[12] Jang N, Choi JE, Kang SH, Bae YK (2019). Validation of the pathological prognostic staging system proposed in the revised eighth edition of the AJCC staging manual in different molecular subtypes of breast cancer. Virchows Arch..

[13] He J, Tsang JY, Xu X, Li J, Li M, Chao X (2020). AJCC 8th edition prognostic staging provides no better discriminatory ability in prognosis than anatomical staging in triple negative breast cancer. BMC Cancer..

[14] Dieci MV, Bisagni G, Brandes AA, Frassoldati A, Cavanna L, Giotta F (2019). Validation of the AJCC prognostic stage for HER2-positive breast cancer in the ShortHER trial. BMC Med.

